# The Effect of Scalp Point Cluster-Needling on Learning and Memory Function and Neurotransmitter Levels in Rats with Vascular Dementia

**DOI:** 10.1155/2014/294103

**Published:** 2014-06-15

**Authors:** Junli Yang, Gerhard Litscher, Haitao Li, Wenhai Guo, Zhang Liang, Ting Zhang, Weihua Wang, Xiaoyan Li, Yao Zhou, Bing Zhao, Qi Rong, Zemin Sheng, Ingrid Gaischek, Daniela Litscher, Lu Wang

**Affiliations:** ^1^Second Affiliated Hospital of Heilongjiang University of Chinese Medicine, Harbin 150001, China; ^2^Research Unit for Complementary and Integrative Laser Medicine, Research Unit of Biomedical Engineering in Anesthesia and Intensive Care Medicine, and TCM Research Center Graz, Medical University of Graz, 8036 Graz, Austria; ^3^Privatclinic Lassnitzhoehe, 8301 Lassnitzhoehe, Austria

## Abstract

We observed the effect of scalp point cluster-needling treatment on learning and memory function and neurotransmitter levels in rats with vascular dementia (VD). Permanent ligation of the bilateral carotid arteries was used to create the VD rat model. A Morris water maze was used to measure the rats' learning and memory function, and the changes in neurotransmitter levels in the rats' hippocampus were analyzed. The results show that scalp point cluster-needling can increase the VD rat model's learning and memory score. The VD rat model's learning and memory score was significantly different when compared with that of the sham operation group (*P* < 0.05). Hippocampal acetylcholine (ACh), dopamine (DA), and 5-hydroxytryptamine (5-HT) concentrations significantly decreased in the rat model. Compared with the model group, the scalp point cluster-needling group's ACh concentration markedly increased and DA and 5-HT levels increased as well. In conclusion, scalp point cluster-needling can improve learning and memory function in VD rats, and its function may be related to an increase in neurotransmitter release.

## 1. Introduction

Vascular dementia (VD) is an acquired intellectual impairment due to impairment in brain function caused by cerebrovascular disease. It is a chronic progressive disease and its main manifestation is changes in learning and memory [1, 2]. As the population gets older and cerebrovascular disease prevalence increases, the occurrence of VD increases as well. In Asian countries, VD accounts for 68.5% of dementia in the older population. In China, it accounts for 60–70%, taking first place [3]. It is predicted that, by 2040, the number of people with dementia worldwide will reach 81.1 million, with the number of dementia patients in China being the sum of those in all the developed countries combined [4]. VD not only severely damages physical and mental health, but also is a serious financial and mental burden on society and the family of those affected. Therefore, it has caught the widespread attention of society and the medical community at home and abroad [5].

Scalp point cluster-needling is a new technique based upon the summation of acupuncture expert Professor Yu Zhishun's many years of clinical experience and is used to treat cerebral infarction. It has been practically proven that scalp point cluster-needling is effective in treating cerebral infarction [6, 7]. The effectiveness of using scalp point cluster-needling to treat VD has yet to be reported. This experiment aims to observe the effect of scalp point cluster-needling on learning and memory function and neurotransmitter levels in rats with VD. Also, this paper will discuss the clinical effectiveness and possible mechanisms of scalp point cluster-needling in treating VD, thereby providing a new train of thought for the prevention and treatment of VD.

## 2. Materials and Methods

### 2.1. Animals

75 Wistar rats (half female, half male, weighing 200–250 g each) were provided by the Heilongjiang University of Chinese Medicine Experimental Animal Center (experimental animal use permit no. SYXK Hei 2008001). They were housed under standard rearing techniques, and room temperature was maintained at 20–25°C. The experiment started after 7 days of adaptive feeding. The investigations were carried out in compliance with the institutional animal care and use committee guidelines.

### 2.2. Main Apparatus

The Morris water maze was manufactured by Anhui Huaibei Zhenghua Biological Instruments and Equipment Ltd., the RF-5301PC fluorescence spectrophotometer by Shimadzu Japan, and the Huatuo brand acupuncture needles (0.30 mm × 13 mm) by Suzhou Medical Appliance Factory Ltd.

### 2.3. Model Preparation

The permanent ligation of bilateral carotid artery method was used to create the VD rat model [8]. Feeding was stopped twelve hours prior to surgery and water was removed 4 hours prior to surgery. A 10% chloral hydrate (3.5 mL/kg) solution was used for intraperitoneal anaesthesia. The rat was laid in a supine position and secured. An incision was made along the midline of the neck, the bilateral common carotid arteries were isolated, a number “0” silk thread was tied around the arteries for ligation, and the wound was closed. During surgery, the rat rectal temperature was maintained at 36.5–37.5°C. After surgery, a penicillin sodium solution was used to coat the local area and then the rats were placed back into their cages. Penicillin sodium was injected intramuscularly at 200 000 U/rat for 3 days continuously.

### 2.4. Model Success Assessment

The sham operation group escape period mean was used as a reference value; if the difference between each VD rat's mean escape latency period and the reference value made up ≥20% of the ratio of the rat's mean escape latency period, then the model was deemed successful [9].

### 2.5. Animal Group Division and Management

The rats were trained in the Morris water maze for two days (after eliminating rats that could not swim or had poor scores) and randomly divided into two groups, 12 in the sham operation group and 40 in the operation group. Then, the surviving rats from the operation group (36 rats) were randomly divided into a model group, cluster-needling group, and positive control group, each containing 12 rats. Treatment was commenced 10 days after surgery, wound healing, and normal feeding.

**Figure 1:**
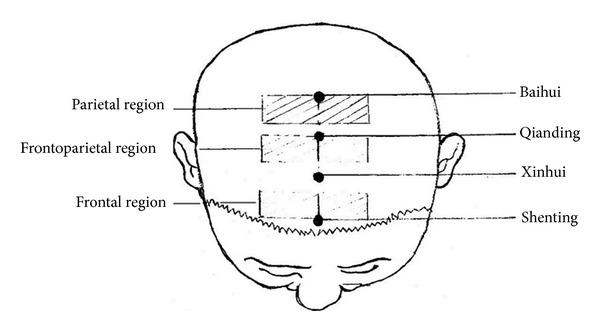
Cluster-needling (note: frontal region: Shenting penetrating Baihui and parallel from Qucha to Benshen. Directly below is the front area of the frontal lobe; parietal region: Baihui penetrating Qianding and 1 cun lateral to right and left Sishencong. Directly below is the precentral gyrus, postcentral gyrus, paracentral lobule, and a portion of the superior parietal lobule and inferior parietal lobule; frontoparietal region: Qianding penetrating Xinghui and Tongtian penetrating Chengguang and Zhengying penetrating Muchuang on both sides. Directly below is the superior frontal gyrus and posterior portion of the middle frontal gyrus).

For the scalp point cluster-needling group, the rats were first fixed on homemade metal racks; the “Laboratory Animal Acupoint Atlas” was used to locate and needle the rat's frontal region, frontal parietal region, and parietal region (cf. human schematic diagram in Figure 1).

In each treatment region, 3 needles were inserted equal distances apart. The needles were retained for 6 hours and stimulated using the twirling method once every hour at a frequency of approximately 200 rotations per minute for 3 minutes. The rats were treated once a day for a total of 4 weeks. In the positive control group, the rats were fixed in the same way as the scalp point cluster-needling group. Baihui and Dazhui acupoints were needled, the needles were retained for 30 minutes, and needles were stimulated every 10 minutes. The frequency of rotation for stimulating Baihui was approximately 200 rotations per minute for 3 minutes, once a day, and it was carried out at the same time as the cluster-needling group. The model group and sham operation group received no acupuncture treatment. An overview of which group received which treatment is shown in Tables 1 and 2.

### 2.6. Observation Indexes and Measurements

*(a) Learning and Memory Function Measurement*. After finishing the treatments, the rats were put into the water maze to test and measure each group's learning and memory function. This included directional navigation training and spatial exploration training. The time required to search for the platform and escape latency (EL) was recorded for 2 minutes. 

*(b) Hippocampus Tissue ACh, DA, and 5-HT Content Measurements.* After the water maze testing was complete, half the rats in each group were euthanized using the spinal cord breaking method, and the brain was quickly removed, with the procedure performed on an ice bed. After the hippocampus was removed and accurately weighed, the tissue was placed in a precooled glass tube and 9 times the physiological saline solution was added to create a 10% brain tissue homogenate. After centrifugation, the clear fluid on top was removed and placed in a −20°C refrigerator to await analyzing. The alkaline hydroxylamine colorimetric method was used to measure the ACh [10, 11] content in the hippocampal tissue, and fluorescence spectrophotometry was used to measure dopamine (DA) and 5-hydroxytryptamine (5-HT) content [12].

### 2.7. Statistical Analysis

**Figure 2:**
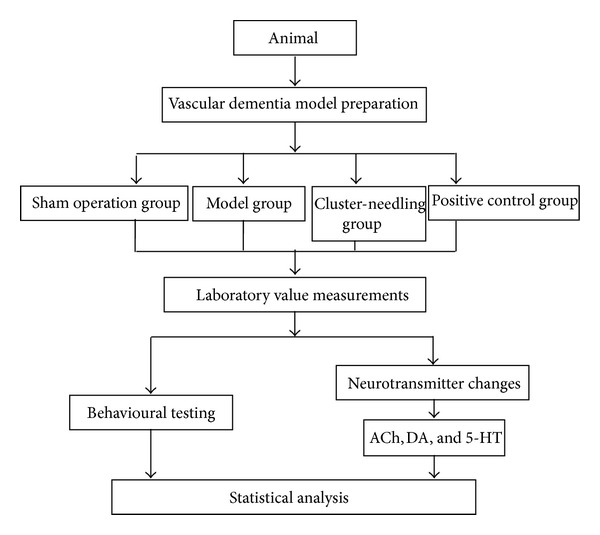
Flowchart of the experimental study.

SPSS 13.0 software was used to perform analysis of variance and the LSD test was used to compare between groups.  Figure 2 shows a flowchart of the experiment in general.

## 3. Results

*(a) Navigation Test*. Four weeks after surgery, each group's (excluding the sham operation group) latency periods were markedly increased and swimming courses were significantly decreased. Escape latencies of rats in the model group and positive control group were significantly longer than that of the sham operation group (*P* < 0.01), indicating that VD rat learning and memory ability was decreased. The scalp point cluster-needling group rats' escape latency period was significantly shorter than that of the model group (*P* < 0.01), indicating that scalp point cluster-needling can improve VD rats' learning and memory function. There was no statistical difference between computational statistical results from the cluster-needling group and sham operation group (*P* < 0.05). This indicates that scalp point cluster-needling can improve learning and memory function in VD model rats (see Table 3).

*(b) Sham Operation Group ACh Concentration Was Significantly Higher Than That of the Model Group* (*P* < 0.01). Compared with the model group, the scalp point cluster-needling group ACh concentration was significantly increased (*P* < 0.01). The ACh concentration of the positive control group was higher than that of the model group; however, the difference was not significant, indicating that scalp point cluster-needling can increase ACh in the VD rat model, and it is more effective than regular acupuncture (see Table 4).

*(c) Hippocampal DA and 5-HF Concentration Comparison between Each Group.* Model rat hippocampal DA and 5-HF concentration were significantly lower than that of the sham operation group (*P* < 0.01). DA and 5-HF concentrations in the positive control group and cluster-needling group were both higher than that of the model group, but the positive control group was not statistically significant. The cluster-needling group had a highly significant difference (*P* < 0.01) and the cluster-needling group had a statistically significant difference when compared to the positive control group (*P* < 0.05). This indicates that scalp point cluster-needling can increase VD rat hippocampal DA and 5-HF levels and its effects are better than that of regular acupuncture (see Table 4).

## 4. Discussion

Scalp point cluster-needling with long retention method was brought about by the acupuncture expert Professor Yu Zhishun [6]. It is based on his many years of clinical experience. Professor Yu Zhishun also created the “needle field” hypothesis. This new scalp acupoint differentiation method integrated field metabolism theory, central nervous system functional location on the scalp, and traditional meridian theory to divide the scalp into 7 treatment regions, including parietal region, frontoparietal region, frontal region, occipital region, lower occipital region, temporal region, and nuchal region. Presently, scalp point cluster-needling is widely used clinically, especially to treat cerebrovascular diseases, where its effectiveness is greater than that of regular scalp needling [13]. However, there is currently no information on the effectiveness of scalp point cluster-needling in treating VD.

VD is caused by cerebrovascular factors which damage brain tissue, but the exact pathogenic mechanism is still unclear. The CA1 area of the hippocampus is where the hippocampus is most closely related to human learning and memory and is easily selectively damaged by ischemia [14]. The normal function of the central cholinergic system is imperative to the formation of memories within the brain, and the cholinergic pathway is learning and memory's main pathway [15]. Within the pathway, ACh is an important neurotransmitter. VD patients' central cholinergic function is markedly decreased, which mainly is manifested in a decrease in ACh activity, causing impairment of learning and memory function [16]. Research has shown that a decrease in monoamine neurotransmitters (including DA, NH, 5-HT, etc.) is closely related to decreases in mental function in VD patients [17–19].

This experiment provides a starting point to look into the effect of scalp point cluster-needling on VD rats' hippocampal neurotransmitter levels, using a currently accepted VD animal model (permanent ligation of bilateral carotid arteries) [20]. It studies the efficacy and possible mechanisms of action of scalp point cluster-needling. The experimental results indicate that while scalp point cluster-needling is improving learning and memory function in VD rats, it also significantly increases hippocampal ACh, DA, and 5-HT concentrations. This indicates that scalp point cluster-needling can improve learning and memory function in VD rats, and one of its mechanisms is related to increasing neurotransmitter concentrations in the brain.

## Figures and Tables

**Table 1:** Different treatment groups and interventions.

	Sham operation group	Model group	Positive control group	Cluster-needling group
Carotid artery ligation	—	X	X	X
Acupuncture at Baihui and Dazhui	—	—	X	—
Scalp point cluster needling	—	—	—	X

**Table 2:** Treatment diagram for each group.

Group	Treatment					
	Times/day	Acupoint selection	Needle retention duration	Duration between needle rotation manipulations	Needle rotating frequency r/min	Rotation manipulation duration
Scalp point cluster-needling group	1	Frontal region, frontoparietal region, and parietal region	6 hours	1 hour	Approx. 200 r/min	3 minutes
Positive control group	1	Baihui, Dazhui	1 hour	10 minutes	Approx. 200 r/min	3 minutes
Model group	None					
Sham operation group	None					

**Table 3:** Comparison of water maze navigation test results for each group (mean ± SD).

Group	Mean escape latency period [s]	Mean swimming distance [cm]
Sham operation group	16.12 ± 6.34∗∗	105.76 ± 16.73∗
Model group	115.36 ± 13.31	174.74 ± 15.81
Positive control group	107.52 ± 16.11	157.65 ± 16.11
Cluster-needling group	62.02 ± 11.61^∗∗△^	124.19 ± 14.25^∗∗△^

Compared with the model group, *P < 0.05 and **P < 0.01; compared with the positive control group, ^△^ *P* < 0.05.

**Table 4:** Hippocampal ACh, DA, and 5-HT measurements for each group (mean ± SD, *n* = 12 per group).

Group	ACh [*μ*g/mg]	DA [ng/g]	5-HT [ng/g]
Sham operation group	27.52 ± 3.13∗∗	521.23 ± 32.54∗∗	652.36 ± 186.13∗∗
Model group	14.39 ± 1.27	362.71 ± 43.15	435.85 ± 152.27
Positive control group	19.05 ± 1.98∗	398.72 ± 34.51∗	525.45 ± 147.33∗
Cluster-needling group	25.82 ± 4.61^∗∗△^	448.63 ± 38.62^∗∗△^	597.59 ± 146.35^∗∗△^

Compared with the model group, *P < 0.05 and **P < 0.01; compared with the positive control group, ^△^ *P* < 0.05.
